# Metabolic disturbances are risk factors for readmission to psychiatric hospitals in non-smokers but not in smokers: results from a Swiss psychiatric cohort and in first-episode psychosis patients

**DOI:** 10.3389/fpsyt.2024.1256416

**Published:** 2024-02-13

**Authors:** Nermine Laaboub, Isabella Locatelli, Claire Grosu, Marianna Piras, Tram Ho Ngoc, Setareh Ranjbar, Martin Preisig, Julien Elowe, Armin von Gunten, Philippe Conus, Chin B. Eap

**Affiliations:** ^1^ Unit of Pharmacogenetics and Clinical Psychopharmacology, Centre for Psychiatric Neuroscience, Department of Psychiatry, Lausanne University Hospital, University of Lausanne, Prilly, Switzerland; ^2^ Centre for Primary Care and Public Health (Unisanté), University of Lausanne, Lausanne, Switzerland; ^3^ Center for Psychiatric Epidemiology and Psychopathology, Department of Psychiatry, Lausanne University Hospital, University of Lausanne, Prilly, Switzerland; ^4^ Service of Adult Psychiatry North-West, Department of Psychiatry, Lausanne University Hospital, University of Lausanne, Prilly, Switzerland; ^5^ Service of Old Age Psychiatry, Department of Psychiatry, Lausanne University Hospital, University of Lausanne, Prilly, Switzerland; ^6^ Service of General Psychiatry, Department of Psychiatry, Lausanne University Hospital, University of Lausanne, Prilly, Switzerland; ^7^ School of Pharmaceutical Sciences, University of Geneva, University of Lausanne, Geneva, Switzerland; ^8^ Center for Research and Innovation in Clinical Pharmaceutical Sciences, University of Lausanne, Lausanne, Switzerland; ^9^ Institute of Pharmaceutical Sciences of Western Switzerland, University of Geneva, University of Lausanne, Geneva, Switzerland

**Keywords:** psychiatry, readmission, relapse, metabolic syndrome, metabolic disturbances, first-episode psychosis

## Abstract

**Background:**

Psychiatric patients are at high risk of readmission, and a high body mass index has previously been shown as a risk factor. We sought to replicate this finding and 1) to prospectively assess the association of metabolic syndrome and its five components with readmission in psychiatric hospitals and 2) to identify other clinical and sociodemographic predictors of readmission.

**Methods:**

Between 2007 and 2019, data on 16727 admissions of 7786 adult and elderly patients admitted to the Department of Psychiatry of the Lausanne University Hospital, were collected. Metabolic syndrome was defined according to the International Diabetes Federation definition. Cox frailty models were used to investigate the associations between readmission and metabolic disturbances.

**Results:**

A total of 2697 (35%) patients were readmitted to our psychiatric hospital. Novel risk factors for readmission in non-smokers were identified, including being overweight (HR=1.26; 95%CI=[1.05; 1.51]) or obese (HR=1.33; 95%CI=[1.08; 1.62]), displaying hypertriglyceridemia (HR=1.21; 95%CI=[1.04; 1.40]) and metabolic syndrome (HR=1.26; 95%CI=[1.02; 1.55]). Central obesity and hyperglycemia increased the risk of readmission when considering the Health of the Nation Outcome Scales variable. In first-episode psychosis patients, obesity (HR=2.23; 95%CI=[1.14; 4.30]) and high-density lipoprotein hypocholesterolemia (HR=1.90; 95%CI=[1.14; 3.20]) doubled the risk of readmission.

**Conclusion:**

The observed interaction between smoking and metabolic variables are compatible with a ceiling effect; metabolic variables increase the risk of readmission in non-smokers but not in smokers who are already at higher risk. Future studies should determine whether better metabolic monitoring and treatment can reduce readmission risk.

## 1 Introduction

Psychiatric disorders are a major public health concern, challenging care providers as well as the entire health system. One in ten individuals globally lives with a psychiatric problem (1), and 38% of Europeans suffer from one each year (2), leading to high costs of illness. In the United States, direct mental health costs reached $225 billion in 2019 (3), while in Switzerland, inpatient mental health care costs nearly 2 billion Swiss francs per year, accounting for approximately 12% of total hospital health expenditures (4). Moreover, in highly developed countries, they are expected to rise from $536 billion in 2010 to an estimated $1.3 trillion in 2030 (5). These costs are either “direct” related to the management of the disease requiring in-and outpatient treatment, or “indirect” related to social impairment (6). Importantly, some psychiatric patients, labeled as “revolving door” or “frequent users” (7), are at high risk of readmission, with an estimated one out of seven patients admitted for psychiatric reasons being readmitted within 30 days of discharge (8). In the United States, readmission rates in a psychiatric hospital range from 11% to 35% (9); in Canada and in the United Kingdom, up to 13% of psychiatric patients are readmitted shortly after discharge (10). In Switzerland, since 2002, the Swiss Health Observatory has documented a 9.3% increase in inpatient admissions in psychiatry and a 30% increase in readmissions, in the context of a reduction of bed numbers and lengths of stay (11). This tendency, which emerged in the frame of the deinstitutionalization movement, raises concerns about the continuity of care following discharge, as well as discharge timing, and makes the early readmission rate a negative indicator of the quality of care (8), which authorities, health organizations, and governments should attempt to reduce. In fact, the identification of readmission risk factors is a necessary first step toward successful control of this phenomenon.

Young age at first admission (12); alcohol, drug, and substance abuse (13); symptoms not in remission at discharge (14); high clinical severity score at discharge (15) and schizophrenia or psychotic symptoms are the key risk factors for readmission in psychiatry (12). Of note, readmission risk increased with the duration and severity of the illness. Therefore, when specifically examining young psychiatric patients experiencing their first episode of psychosis, duration of untreated psychosis, comorbid substance use disorders, a low Global Assessment of Functioning score, and poor treatment adherence were found to increase the likelihood of readmission (16–18). Interestingly, recent research has tended to evaluate the impact of physical comorbidities, especially metabolic ones, on increasing the likelihood of readmission, which remains a matter of debate (8, 19). A recent study of 945 patients found that a high body mass index [median BMI=28.5 Kg/m (2)] triples the readmission risk in psychiatry (19). Nevertheless, to our knowledge, these results have not yet been replicated, while the relationship between the other metabolic disturbances (e.g., dyslipidemia) and readmission have not considered all psychiatric and first-episode psychosis patients separately. For instance, elevated lipid levels are associated with smaller brain structures, more severe mood symptoms and cognitive dysfunction, poorer sleep quality, and increased impulsivity, all of which can lead to relapse and readmission (20). Besides, psychiatric patients face a significantly heightened susceptibility to metabolic disturbances (21), resulting in a decrease in life expectancy exceeding 10 years (22). This can be attributed to the use of psychotropic drugs, particularly antipsychotics (23), along with other reasons, notably smoking habits, sedentary lifestyle, and unhealthy diets (24). Considering the high prevalence of readmission and metabolic disturbances in the psychiatric population, additional investigation is required to identify and characterize the relationship between both factors.

In a large Swiss psychiatric cohort with a 13-year follow-up, this study aims to prospectively assess the association of metabolic disturbances, namely high BMI, central obesity, hyperglycemia, hypertriglyceridemia, high-density lipoprotein (HDL) hypocholesterolemia, hypertension, and metabolic syndrome, with time to readmission in psychiatric hospital, as well as to identify other sociodemographic predictors of readmission. In addition, as readmission rate increases with the severity of the illness, any association between metabolic disturbances and readmission could be attributed to extended duration of illness and increased period of prescriptions of psychotropic drugs that risk metabolic disturbances. We aimed to replicate the findings in a subgroup of first-episode psychosis patients.

## 2 Methods

### 2.1 Study design, setting and participants

In 2007, the Department of Psychiatry at the University Hospital of Lausanne established a guideline for the monitoring of patients who were starting or continuing psychotropic treatment with a potential risk of inducing metabolic disorders (listed in **Supplementary Table 1**). Since then, a longitudinal observational study (PsyMetab) has been running using this guideline (25). As a result, patients who provided informed consent for the PsyMetab study or accepted the general consent of the Lausanne University Hospital were included. Furthermore, the canton of Vaud’s ethics committee (CER-VD) has approved the use of clinical data from patients followed between 2007 and 2015 without informed consent due to the non-interventional *post-hoc* analysis study design (PsyClin). First-episode psychosis patients, patients with the shortest duration of illness of the whole cohort, with no or minimal history of prescription of psychotropic drugs for no longer than 6 months, were recruited from the Treatment and Early Intervention in Psychosis Program (TIPP) (26) and the PsyMetab study. Patients included in the TIPP cohort gave their written informed consent, and the TIPP protocol was approved by CER-VD. The current study included observations concerning adult and elderly patients collected between January 1, 2007, and December 31, 2019.

### 2.2 Data extraction

Data were retrieved from electronic administration and prescription hospital databases. Age, sex, marital status, education, living situation, employment, and admission status (voluntary or compulsory) were among the sociodemographic and clinical data collected. In order to calculate the Swiss socioeconomic position (SSEP, a postal-address-based metric ranging from 0 (most disadvantaged) to 100 (most privileged) that determines the socioeconomic status of Swiss residents based on postal address), patient addresses were extracted and geocoded using the Google API and the ggmap R package (27, 28). Clinical severity at discharge was assessed using the Health of the Nation Outcome Scales (HoNOS; an instrument comprising 12 simple scales measuring behavior, impairment, symptoms, and social functioning) (29). Smoking status was established based on self-reported tobacco consumption, the presence of ICD-10 mental and behavioral disorders due to the use of tobacco, and/or the use of nicotine or varenicline. Suicide attempts were based on the presence of ICD-10 X84.9 intentional self-harm by unspecified means. Psychiatric diagnoses were based on ICD-10 codes and were classified into seven diagnostic groups as follows: dementia [F00-F02 and G30], substance use disorders [F10-F19], psychotic disorders [F20-F24 and F28-F29], schizoaffective disorders [F25], bipolar disorders [F30-F31], depression [F32-F33], and other diagnoses [F03-F09 and F34-F99 (e.g., eating disorders or nonorganic sleep disorders); used as reference category in models]. Psychotropic medications were classified by their weight-gain risk (**Supplementary Table 1**), whereas somatic comorbidities were derived from ICD-10 and supplemented with ATC codes and/or laboratory test results (**Supplementary Table 2**). BMI was calculated by dividing weight in kilograms by height in square meters. According to the International Diabetes Federation (IDF) definition, laboratory values and ATC codes were used to detect the presence of metabolic syndrome and its five components (central obesity, hyperglycemia, hypertriglyceridemia, HDL hypocholesterolemia, and hypertension; **Supplementary Tables 3A, B**) (30). The information is gathered prospectively, resulting in repeated measurements per stay. As a starting point for follow-up for the next hospital stays, the last measurements of the previous hospital stays were used.

### 2.3 Data analysis

Using Pearson’s χ2 and Wilcoxon tests, readmitted in our psychiatric hospital (at least one additional admission) and non-readmitted (only one admission) patients were first compared in terms of baseline sociodemographic, clinical, and metabolic features. For all patients, from the initial hospital discharge after January 1, 2007, we recorded each out-of-hospital period. This resulted in repeated measures of duration data, which was right-censored if the patient was not in the hospital at the end of follow-up on December 31, 2019. Univariate analyses were performed using Cox frailty models to identify predictors of readmission risk and investigate the eventual time-dependence of their effect (proportionality assumption) (31, 32).

Different multivariable models were built, including in turn the presence of metabolic syndrome and each metabolic disturbance (hypertriglyceridemia, HDL hypocholesterolemia, hyperglycemia, hypertension, central obesity, and BMI). Each of these models was adjusted to include factors showing a significant association with readmission risk in the univariate setting (p ≤0.1) and having less than 60% of missing values in order to keep a sufficiently large sample size. Interactions between risk factors and time were kept in the models only when they showed statistical significance (p ≤0.05). Models were further adjusted for the interaction between smoking and metabolic disturbances, as significant associations between the two had previously been reported (33–36). Finally, the analyses were replicated in a subset of first-episode psychotic patients.

Data preparation and univariate and multivariable analyses were performed using STATA version 16.0 for Windows (StataCorp, Texas, USA) and R version 4.1.1 for Windows (GNU General Public License version 2, Massachusetts, USA).

## 3 Results

### 3.1 Baseline cohort characteristics

Between January 1, 2007, and December 31, 2019, a total of 16727 admissions occurred, which involved the 7786 patients who were included in the current study. These included 2,697 (35%) patients were readmitted; half of them were readmitted 3 times (interquartile range=1-9) with a maximum number of readmissions of 80. **Tables 1**–**3** display the baseline sociodemographic, clinical, and metabolic characteristics of the study population. Readmitted patients were younger than non-readmitted patients and had a lower SSEP (**Table 1**). Marital status was associated with readmission status but not sex, education, employment, or living situation. Smokers were more likely to be readmitted and the distribution of psychiatric disorders and psychotropic medication use (classified by their risk of weight-gain) differed between readmitted and non-readmitted patients (**Table 2**). Readmitted patients were less likely to be affected with cardiovascular disease, whereas no significant difference was observed for other somatic comorbidities, suicide attempts, nor for HoNOS and admission status. Readmitted patients had a higher BMI and included a higher proportion of obese patients. Trends toward a lower proportion of underweight patients, of patients with normal BMI and of HDL hypocholesterolemia were observed in the readmitted group (**Table 3**).

**Table 1:** Baseline demographic characteristics of the readmitted and non-readmitted patients.

Characteristics	Total sample 7786 (100%)	Readmission		p value
		No 5089 (65%)	Yes 2697 (35%)	
**Age**, median (IQR), years	47 (32;70)	50 (34;76)	43 (30; 58)	**<10^-4^**
**Sex**, n (%) Men Women	3659 (47) 4127 (53)	2381 (47) 2708 (53)	1278 (47) 1419 (53)	0.61
**Marital status**, n (%) Married or registered partnership Single Divorced or separated Widowed	1844 (24) 3051 (40) 1882 (24) 942 (12)	1354 (27) 1785 (35) 1146 (23) 741 (15)	490 (18) 1266 (47) 736 (27) 201 (7)	**<10^-3^**
**Education**, n (%) Compulsory schooling No schooling Apprenticeship High school University or college	141 (26) 30 (6) 175 (33) 45 (8) 142 (27)	99 (26) 24 (6) 123 (33) 33 (9) 96 (26)	42 (27) 6 (4) 52 (33) 12 (8) 46 (29)	0.72
**Employment**, n (%) Employed Unemployed Pensions [disability, retirement] Other [e.g., student]	164 (20) 224 (27) 383 (47) 47 (6)	123 (21) 152 (26) 272 (47) 35 (6)	41 (17) 72 (31) 111 (47) 12 (5)	0.45
**Living situation**, n (%) Home, with others Home, alone Homeless Institutions [(non) medico-social] Other [prisons, psychiatric clinics, other establishments]	457 (52) 279 (31) 19 (2) 97 (11) 34 (4)	338 (54) 187 (30) 15 (2) 62 (10) 26 (4)	119 (46) 92 (36) 4 (2) 35 (13) 8 (3)	0.11
**Swiss socio-economic position** median (IQR)	57 (46; 65)	57 (46; 66)	56 (45; 65)	**0.002**

Pearson’s χ2 test was employed to detect the intergroup difference in terms of categorical variables, while Wilcoxon rank-sum test was used for the continuous variables.

The total n value differs between variable due to missing data.

Number of observations without missing data: age: n=7786 (% of missing values: 0%); sex: n=7786 (0%); marital status: n=7719 (<1%); education: n=533 (93%); employment: n=818 (90%); living situation: n=886 (89%); Swiss socio-economic position: n=5134 (34%).

IQR, interquartile range; n, number.

Statistically significant p-values in bold.

**Table 2:** Baseline clinical characteristics of the readmitted and non-readmitted patients.

Characteristics	Total sample 7786 (100%)	Readmission		p value
		No 5089 (65%)	Yes 2697 (35%)	
**Psychiatric disorders**, n (%) Dementia Substance use disorders Psychotic disorders Schizoaffective disorders Bipolar disorders Depressive disorders Other	742 (11) 1397 (20) 1654 (24) 305 (4) 686 (10) 1751 (25) 427 (6)	639 (15) 833 (19) 867 (20) 122 (3) 401 (9) 1134 (26) 350 (8)	103 (4) 564 (21) 787 (30) 183 (7) 285 (11) 617 (24) 77 (3)	**<10^-3^**
**Psychotropic medication by risk of weight gain**, n (%) No risk Low risk Medium risk High risk	6496 (84) 265 (3) 711 (9) 314 (4)	4350 (86) 152 (3) 426 (8) 161 (3)	2146 (79) 113 (4) 285 (11) 153 (6)	**<10^-3^**
**Smokers**, n (%) No Yes	1514 (43) 1970 (57)	836 (53) 727 (47)	678 (35) 1243 (65)	**<10^-3^**
**Admission status**, n (%) Voluntary Compulsory	509 (56) 402 (44)	357 (55) 291 (45)	152 (58) 111 (42)	0.45
**Suicide attempt**, n (%) No Yes	6947 (>99) 15 (<1)	6270 (>99) 15 (<1)	677 (100) 0 (0)	0.21
**HoNOS**, median (IQR)	11 (6;16)	10 (6;16)	11 (6;16)	0.16
**Somatic comorbidities**, n (%) Diabetes Cardiovascular diseases COPD Thyroid dysfunction Hypothyroidism Hyperthyroidism	445 (6) 70 (0.9) 23 (<1) 106 (1) 25 (<1)	297 (6) 55 (1.1) 17 (<1) 71 (1) 18 (<1)	148 (5) 15 (0.6) 6 (<1) 35 (1) 7 (<1)	0.52 **0.02** 0.39 0.74

Pearson’s χ2 test was employed to detect the intergroup difference in terms of categorical variables, while Wilcoxon rank-sum test was used for the continuous variable.

The total n value differs between variables due to missing data.

Number of observations without missing data: psychiatric disorders: n=6962 (% of missing values: 11%); psychotropic medication: n=7786 (0%); smokers: n=3484 (55%); somatic comorbidities: n=7786 (0%); admission status: n=911 (88%); suicide attempts: n=6962 (11%); HoNOS score: n=2134 (73%).

Psychiatric diagnoses were based on the ICD-10 classification and were classified as: dementia [F00-F02; G30] | substance use disorders [F10-F19] | psychotic disorders [F20-F24; F28-F29] | schizoaffective disorders [F25] | bipolar disorders [F30-F31] | depression [F32-F33] | others [F03-F09; F34-F99].

Psychotropic medication was classified according to the risk of weight gain as follows: low risk: amisulpride, aripiprazole, brexpiprazole, chlorprothixene, clotiapine, cariprazine, doxepin, flupentixol, haloperidol, lurasidone, opipramol, pipamperone, promazine, sertindole, sulpiride, tiapride; medium risk: amitriptyline, asenapine, carbamazepine, clomipramine, levomepromazine, lithium, mirtazapine, nortriptyline, pregabalin, paliperidone, quetiapine, risperidone, trimipramine, zuclopenthixol; high risk: clozapine, olanzapine, valproate.

COPD, chronic obstructive pulmonary disease; HoNOS, Health of the Nation Outcome Scales; IQR, interquartile range; n, number.

Statistically significant p-values in bold.

**Table 3:** Baseline metabolic characteristics of the readmitted and non-readmitted patients at discharge.

Characteristics	Total sample 7786 (100%)	Readmission		p value
		No 5089 (65%)	Yes 2697 (35%)	
**BMI**, median (IQR), kg.m^-2^	23.9 (21.0; 27.6)	23.6 (20.9; 27.3)	24.2 (21.1; 28.3)	**0.0003**
A. **BMI categories**, n (%) • Normal • Underweight • Overweight • Obese	1632 (50) 284 (9) 803 (25) 510 (16)	975 (52) 180 (10) 463 (25) 262 (14)	657 (49) 104 (8) 340 (25) 248 (18)	0.08 0.07 0.71 **0.001**
B. **Central obesity**, n (%), IDF definition	650 (20)	361 (19)	289 (21)	0.11
C. **Hypertriglyceridemia**, n (%)	1474 (35)	1029 (35)	445 (34)	0.64
D**. HDL hypocholesterolemia**, n (%)	1765 (38)	1225 (39)	540 (36)	0.052
E. **Hypertension**, n (%)	2127 (78)	1580 (77)	547 (78)	0.96
F. **Hyperglycemia**, n (%)	1526 (28)	1010 (28)	516 (26)	0.12
G. **Metabolic syndrome**, n (%), IDF definition	339 (9)	208 (8)	131 (9)	0.45

Pearson’s χ2 test was employed to detect the intergroup difference in terms of categorical variables, while Wilcoxon rank-sum test was used for the continuous variable.

The total n value differs between variables due to missing data.

Number of observations without missing data: BMI: n=3229 (% of missing values: 59%); central obesity: n=3248 (58%); hypertriglyceridemia: n=4237 (46%); HDL hypocholesterolemia: n=4653 (40%); hypertension: n=2744 (65%); hyperglycemia: n=5527 (29%); metabolic syndrome: n=3886 (50%).

A. Defined according to the World Health Organization (WHO) definition: Normal weight (reference): 18.5≤ body mass index (BMI) ≤ 25; Underweight: BMI <18.5 kg.m^-2^; Overweight: 25 ≤ BMI ≤ 30; Obese: BMI>30.

B. Defined using the IDF definition as follows: waist circumference: men ≥ 94 cm; women ≥ 80 cm; or BMI > 30 kg.m^-2^.

C. Defined as follows: systolic BP ≥ 130 or diastolic BP ≥ 85 mm Hg or treatment for hypertension.

D. Defined as follows: fasting plasma glucose ≥ 5.6 mmol/L or treatment for type 2 diabetes.

E. Defined as follows: HDL cholesterol: men < 1.03 mmol/L; women < 1.29 mmol/L or treatment for lipid abnormality.

F. Defined as follows: triglycerides ≥ 1.7 mmol/L or treatment for lipid abnormality.

G. Defined using the International Diabetes Federation (IDF) definition as follows: presence of the B factor plus any two of the following: C/D/E and/or F factors.

BMI, body mass index; HDL, high-density lipoprotein; IQR, interquartile range; kg, kilograms; m^-2^, square meter; n, number.

Statistically significant p-values in bold.

Given the high number of missing values (>80%) in the education, employment, living situation, and admission status variables and the few numbers of patients with somatic comorbidities (<6%), those variables were excluded from further analyses. Of note, although the variable HoNOS contains almost 65% of missing values, as it has previously been associated with readmission risk, sensitivity analyses were performed considering this variable.

### 3.2 Metabolic and other factors associated with readmission in the whole cohort

A total of 16580 out-of-hospital periods were recorded, with the number of periods differing between models due to missing data. Cox frailty univariate models revealed that female sex, age≥45 years, high SSEP, dementia, and hypertension were all associated with a reduced risk of readmission in psychiatry. Conversely, smoking; substance abuse; psychotic, schizoaffective, bipolar, and depressive disorders; a high HoNOS and a high BMI at previous discharge were associated with increased risk of readmission (see **Supplementary Table 4** for more detail). Psychotropic medication, suicide attempts, central obesity, hyperglycemia, hypertriglyceridemia, HDL hypocholesterolemia, and metabolic syndrome were not associated with readmission. Sex, age, HoNOS, hypertension, and metabolic syndrome were all found to have a time-dependent effect.

**Figure 1:**
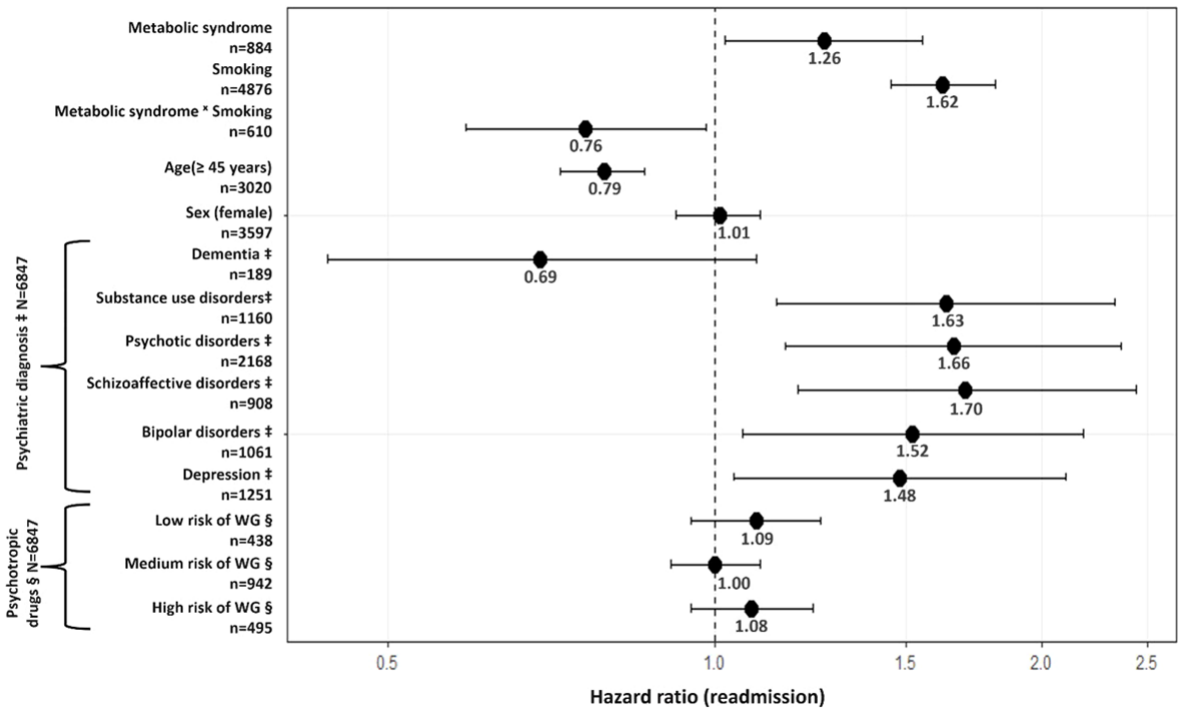
Association between metabolic syndrome and psychiatric readmission. N=6847 observations in the model. n=number of observations in the variable. ‡Compared to other diagnoses (e.g., anxiety disorders). § Compared to No risk of WG. Psychiatric diagnoses were based on the ICD-10 classification and were classified as: dementia [F00-F02; G30] | substance use disorders [F10-F19] | psychotic disorders [F20-F24; F28-F29] | schizoaffective disorders [F25] | bipolar disorders [F30- F31] depression [F32-F33] | others [F03-F09; F34-F99]. Psychotropic medication was classified according to the risk of weight gain as follows: low risk: amisulpride, aripiprazole, brexpiprazole. chlorprothixene, clotiapine, cariprazine, doxepin, flupentixol, haloperidol, lurasidone, opipramol, pipamperone, promazine, sertindole, sulpiride, tiapride; medium risk: amitriptyline, asenapine, carbamazepine, clomipramine, levomepromazine, lithium, mirtazapine, nortriptyline, pregabalin, paliperidone, quetiapine, risperidone, trimipramine, zuclopenthixol; high risk: clozapine, olanzapine, valproate. Of note, sensitivity analyses were performed to account for borderline personality disorder (F60.3) subgroup diagnosis (number of observations with borderline personality disorder included in models ranged between 307 and 681). However, no significant association was observed between borderline personality disorder and readmission in our cohort. WG, weight gain.

**Figure 2:**
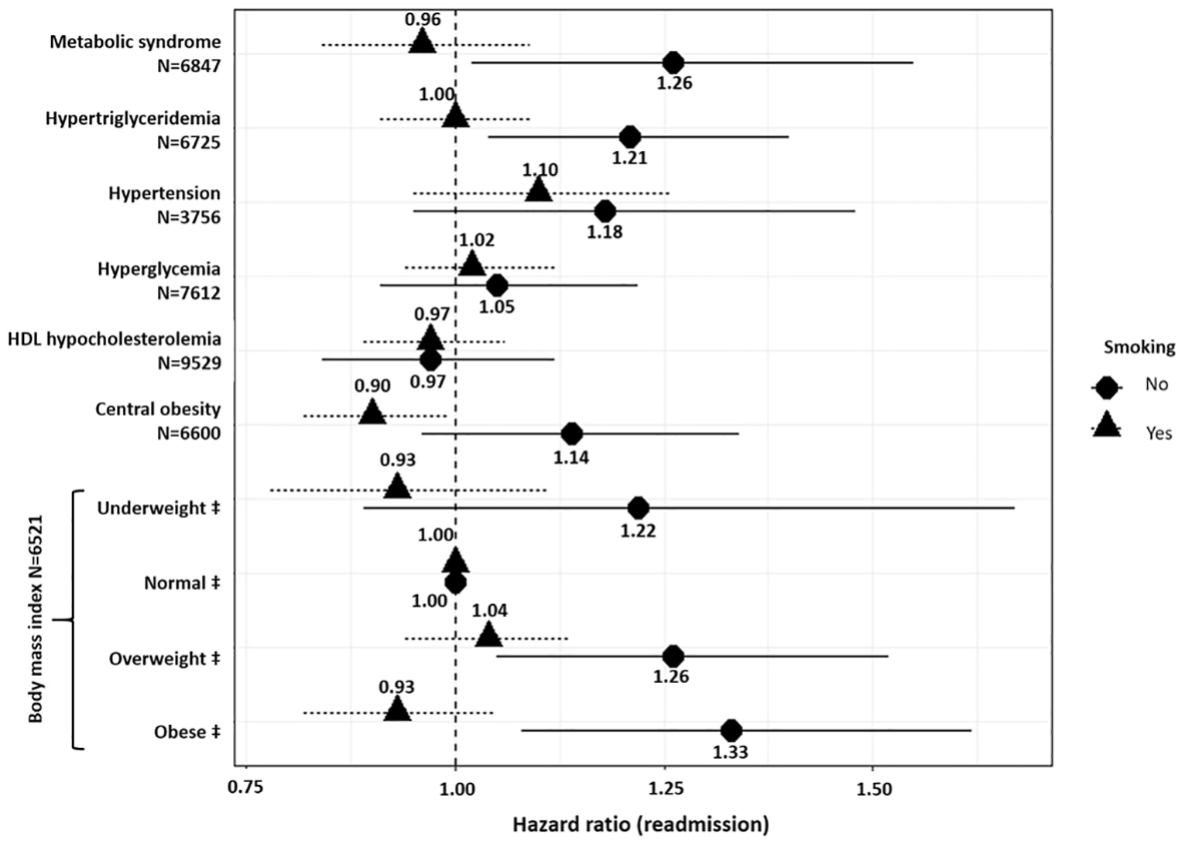
Association between metabolic disturbances and psychiatric readmission. ‡ Compared to normal weight and defined as follows: Normal weight (reference): 18.5≤ body mass index (BMI) ≤ 25; Underweight: BMI <18.5 kg.m^-2^; Overweight: 25 ≤ BMI ≤ 30; Obese: BMI>30. Models were adjusted for age, sex, smoking status, psychiatric diagnoses, psychotropic medication, and interaction between the metabolic disturbance analyzed and smoking. N: number of observations in each model. N varies due to missing values.

A first Cox frailty model including metabolic syndrome with adjustments for variables associated with readmission according to the univariate Cox frailty models and a lenient significance level of p<0.1, provided a significant interaction between metabolic syndrome and smoking status (p=0.03). This indicates significant differences between smokers and nonsmokers in the strength of the association between metabolic syndrome and readmission. Indeed, among non-smokers, metabolic syndrome raises the risk of readmission in psychiatry by 26% (95% confidence interval (95% CI)=[1.02; 1.55]; **Figure 1**). However, metabolic syndrome does not raise the risk of readmission among smokers (HR=0.96; 95%CI=0.84; 1.09; **Figure 2**), who are already at a higher risk of readmission as compared to non-smokers (HR=1.62; 95%CI=[1.45; 1.81]; **Figure 1**). In addition, no significant violation of the proportionality assumption was detected in the multivariable setting; hence, all interactions of covariates with time were dropped from the model. Finally, several demographic and clinical predictors of readmission in psychiatry were also identified. Thus, being age over 45 years was significantly related to a decreased risk of readmission (**Figure 1**). Conversely, being diagnosed with substance abuse or psychotic, schizoaffective, bipolar, or depressive disorders raised the risk of readmission as compared to other disorders (e.g., anxiety, mental retardation) (**Figure 1**). Psychotropic medication and sex were not associated with readmission.

**Figure 2** provides the results of Cox frailty models including each metabolic disturbance variable as a potential predictor of readmission in psychiatry. Models were adjusted for the interaction between each metabolic disturbance and smokingstatus. In non-smokers, being underweight (BMI <18.5 kg/m^2^), overweight (25 kg/m^2^ ≤ BMI ≤30 kg/m^2^), or obese (BMI > 30 kg/m^2^) was associated with 22% (95%CI=[0.89; 1.67]), 26% (95%CI=[1.05; 1.51]), and 33% (95%CI=[1.08; 1.62]) increased risk of readmission, respectively, whereas this was not the case in smokers. In addition, hypertriglyceridemia increased the risk of readmission by 21% (95%CI=[1.04; 1.40]) in non-smokers. Hypertension, hyperglycemia, central obesity, and HDL hypocholesterolemia were not associated with risk of readmission regardless of smoking status.

### 3.3 Sensitivity analyses in the whole cohort

**Supplementary Figure 1** provides the results of sensitivity analyses performed by introducing the HoNOS variable into the Cox frailty multivariable models (number of observations between 2773 and 3535, depending on the model considered). Hence, a significant association was found between elevated HoNOS at previous discharge and the risk of readmission regardless of the metabolic disturbance considered (HR ≥ 1.12; 95%CI= [1.07; 1.16] for each 5-unit increase, data not shown). In addition, comparable results for the association between metabolic disturbances and readmission were found, except for the effects of central obesity and hyperglycemia on the risk of readmission becoming significant in non-smokers (HR _central obesity_= 1.46; 95% CI=[1.17; 1.84]; HR _hyperglycemia_= (HR=1.34; 95% CI=[1.05; 1.72])).

### 3.4 Metabolic factors associated with readmission in first-episode psychosis patients (subgroup analyses)

**Supplementary Tables 5** and **6** display the baseline sociodemographic, clinical, and metabolic characteristics of first-episode psychosis patients (N=285). Except for the distribution of living situation and admission status, no significant difference was observed between readmitted and non-readmitted patients. Of note, two (2%) and 11 (6%) of non-readmitted and readmitted patients, respectively, had concomitant substance use disorder diagnosis (p=0.26), data not shown).

A total of 1084 out-of-hospital periods were recorded; however, the presence of missing data resulted in variations in the number of periods across models. **Supplementary Figure 2** includes multivariable analyses of first-episode patients (considering smokers and non-smokers; number of observations between 270 and 650, depending on the model considered). In non-smokers, being obese doubled the risk of readmission (HR= 2.23; 95%CI=[1.14; 4.33]), while HDL hypocholesterolemia raised this risk by 90% (95%CI=[1.14; 3.19]). Regarding BMI (under- or overweight), central obesity, hypertriglyceridemia, hyperglycemia, hypertension, and metabolic syndrome, none reached the threshold of statistical nbsp;significance. Moreover, in smokers, no association was found between obesity, central obesity, hypertriglyceridemia, hyperglycemia, and the risk of readmission. Nevertheless, overweight smoking patients tended to have 33% (95%CI=[0.98; 1.79]) additional risk of readmission, whereas smoking patients with metabolic syndrome were at 54% decreased risk of readmission (95%CI=[0.25; 0.83]). Finally, sensitivity analyses in first-episode psychosis patients considering the HoNOS variable showed no significant relationship between any of the metabolic disturbances and readmission either in smokers or in non-smokers (number of observations between 255 and 325, depending on the model considered; **Supplementary Figure 3**).

## 4 Discussion

The present study, with a readmission rate similar to other studies (37–39), is the first to show, in a large cohort of patients with a 13-year follow-up, that central obesity, hypertriglyceridemia, hyperglycemia and metabolic syndrome are independent predictors of readmission in non-smokers but not in smokers. This study is also the first to confirm that a high BMI increases the risk of readmission. Overweight and obese non-smoking patients had a 26% and 33% extra risk of readmission, respectively, confirming prior findings (19). Our study jointly showed that psychotic, schizoaffective, and bipolar patients and those whose management requires the prescription of psychotropic drugs (e.g., antipsychotics or mood stabilizers) known to induce weight gain are at significant risk of readmission (23). Thus, illness severity may relate high BMI to readmission. In particular, severe symptoms and/or resistance to treatment increase the likelihood of psychotropic drug combinations, which can lead to weight gain and/or metabolic disorders. This, in turn, can affect the patient’s perception of their body, inducing non-adherence and relapse. Of note, at baseline, readmitted patients had higher BMIs and a higher proportion were obese compared to non-readmitted patients. It is in agreement with results showing that psychiatric illness is associated with metabolic worsening independent of psychotropic drug use (40). Importantly, after restricting the analysis to patients with a first-episode of psychosis and adjusting for psychotropic drugs, obesity doubled the probability of readmission, suggesting a possible direct role of obesity in relapse and readmission. Of note, inflammation may serve as a mediator in the relationship between elevated BMI and readmission. Inflammation is linked to both obesity and psychiatric disorders (41, 42), as well as the severity of illness (43). One study reported that elevated CRP levels were associated with an increased risk of depressive relapse (44), and a strong association between inflammatory processes and recurrent major depressive disorders has been suggested (45).

Central obesity tended to increase the risk of readmission among non-smokers in the whole cohort and in first-episode psychosis patients, the association being statistically significant when considering HoNOS for the entire sample. This could be explained by the fact that HoNOS is more likely to be used for severe cases (HoNOS was available for less than 35% of our patients). On the other hand, this may be a result of the association between high BMI and readmission risk, as centrally obese patients had significantly higher BMI than non-centrally obese patients. In addition, central obesity is linked to stress, anxiety, inflammation, psychosis, and depression (46–48), which are common in psychiatric patients.

Our results revealed an increased risk of readmission in non-smoking patients suffering from hyperglycemia when considering HoNOS. Non-smokers with first-episode psychosis had similar effects, although the small sample size precluded statistical significance. Recent studies found that the disruption of glucose-insulin homeostasis may presage psychosis (49), and that insulin resistance is related to pre-psychotic symptoms in young individuals before the onset of clinical psychosis (50). One case report linked hyperglycemia to psychotic symptoms, and a case series showed that poor blood glucose management may have been connected to psychosis (51, 52), particularly as increased insulin is associated with mood alteration (53). Psychiatric patients have a high prevalence of hyperglycemia and diabetes, late diagnosis, poor somatic management (54, 55) and non-adherence to somatic treatment (56), which may lead to psychosis or relapse and readmission.

For the first time, hypertriglyceridemia is identified as a significant independent predictor of readmission in non-smokers. Psychotropic drug use, a poor diet, and/or sedentary lifestyle can induce metabolic abnormalities including hypertriglyceridemia (57). In addition, obesity, which independently predicts readmission, is associated with high levels of triglycerides. Of note, high triglyceride level is a significant marker for readmission for deliberate self-harm (58), and is associated with generalized anxiety disorder (59). On the other hand, high triglycerides and C-reactive protein levels at baseline have been linked to unfavorable clinical outcomes and a decreased rate of treatment response at one-year follow-up (60), and a second study identified an interaction between lipids and inflammation as a predictor of worse negative symptom severity in patients with schizophrenia, which suggests a mediating role of inflammation in the association between hypertriglyceridemia and readmission.

HDL hypocholesterolemia was not associated with readmission when the entire cohort was considered, regardless of HoNOS. Nevertheless, first-episode psychosis non-smoking patients with HDL hypocholesterolemia had a 90% higher risk of readmission. These findings should be carefully evaluated due to the small sample size. A recent study found a substantial link between dyslipidemia and acute-phase schizophrenia (61). Thus, more mechanistic studies are needed to better understand the relationship between HDL hypocholesterolemia and readmission in psychiatry.

No significant correlation between hypertension and readmission was identified. The high prevalence of hypertension in the elderly may account for this finding. Considering associations between hypertriglyceridemia, central obesity, hyperglycemia, and readmission, it was not surprising to find an increased risk of readmission in non-smoking patients with metabolic syndrome. Thus, inpatients with severe mental illness whose metabolic risk factors were poorly monitored had an increased risk of readmission (62).

The prevalence of smoking is notably higher among those with severe mental illness (63). In addition, in patients with schizophrenia, bipolar disorder, or major depression, smoking increases the likelihood of being admitted to a psychiatric hospital by 258% (64). Our study revealed that smokers had a higher readmission risk than non-smokers, regardless of metabolic abnormalities. Although smoking lowers hip fat accumulation (65), current smokers are more likely to be centrally obese than never smokers (34) and nicotine is likely to mediate the majority of the effect of smoking on body weight and fat deposits (66). In addition to the aforementioned effects on metabolic parameters, smoking also causes alterations in insulin secretion and may promote insulin resistance (67), as well as increases triglycerides and decreases HDL cholesterol levels (35). Thus, the link between metabolic disturbances and readmission in psychiatry among smokers needs to be further investigated. According to earlier research, a first admission at a young age strongly predicts readmission (68, 69), as well as the clinical severity measured by HoNOS (15, 70). Indeed, high HoNOS at discharge in the readmitted group may indicate premature discharge and poor recovery. Finally, the present study found no gender difference in readmission risk.

Our study has several limitations. First, we cannot rule out the possibility of readmission to other psychiatric hospitals or death prior to readmission in psychiatry. Consequently, readmission rates might be underestimated. Second, missing data were common in several variables leading to the inclusion of only a subset of patients when such variables were considered. Only associations were determined, and no causal relationship could be identified due to the study design. Finally, observed associations might be mediated by factors not available and/or identified (i.e., suicide risk, antisocial traits) and further research exploring the nature of these associations is warranted. Despite these limitations, the present results strongly support routine consideration of metabolic disturbances by clinicians in order to improve patient management.

## 5 Conclusion

Hypertriglyceridemia, high BMI, hyperglycemia, central obesity, and metabolic syndrome were identified as independent predictors of readmission in psychiatry in non-smokers but not in smokers. Similar results were found in first-episode psychosis patients whose obesity and HDL hypocholesterolemia doubled the risk of readmission. Future studies should examine whether a causal relationship can be identified and demonstrate whether better monitoring of metabolic parameters may contribute to reducing the risk of readmission to a psychiatric hospital.

## Data availability statement

Due to the sensitivity of the data and the absence of informed consent for public data depository, the datasets analyzed during this study are not available to the public. The dataset supporting the conclusions of this article were obtained from PsyMetab study. Requests to access the datasets should be directed to: research.psymetab@chuv.ch.

## Ethics statement

The studies involving humans were approved by the Ethics committee of Vaud (CER-VD). The studies were conducted in accordance with the local legislation and institutional requirements. The participants provided their written informed consent to participate in this study.

## Author contributions

NL: Data curation, Formal analysis, Investigation, Methodology, Writing – original draft. IL: Formal analysis, Methodology, Supervision, Validation, Writing – review & editing. CG: Data curation, Writing – review & editing. MPi: Data curation, Writing – review & editing. TN: Data curation, Writing – review & editing. MPr: Methodology, Supervision, Validation, Writing – review & editing. JE: Validation, Writing – review & editing. AG: Supervision, Validation, Writing – review & editing. PC: Funding acquisition, Supervision, Validation, Writing – review & editing. CE: Funding acquisition, Investigation, Project administration, Resources, Supervision, Validation, Visualization, Writing – review & editing. SR: Formal analysis, Methodology, Supervision, Validation, Writing – review & editing.
