# Supplementary material for: Metabolic disturbances are risk factors for readmission to psychiatric hospitals in non-smokers but not in smokers: results from a Swiss psychiatric cohort and in first-episode psychosis patients
Source: Front Psychiatry. 2024 Feb 13;15:1256416. doi: 10.3389/fpsyt.2024.1256416 (PMC10896922; doi:10.3389/fpsyt.2024.1256416)
Supplement: Supplementary file 1 [file DataSheet_1.docx]

**Metabolic disturbances are risk factors for readmission to psychiatric hospitals in non-smokers but not smokers: results from a Swiss psychiatric cohort and in first-episode patients**

**Supplementary files**

**Supplementary Table 1:** Psychotropic medications categorized by risk of weight gain

| **High risk** | **Medium risk** | **Low risk** | **No risk** |
| --- | --- | --- | --- |
| Clozapine | Amitriptyline | Amisulpride | All the other psychotropic medications |
| Olanzapine | Asenapine | Aripiprazole |  |
| Valproate | Carbamazepine | Brexpiprazole |  |
|  | Clomipramine | Chlorprothixene |  |
|  | Levomepromazine | Clotiapine |  |
|  | Lithium | Cariprazine |  |
|  | Mirtazapine | Doxepin |  |
|  | Nortriptyline | Flupentixol |  |
|  | Pregabalin | Haloperidol |  |
|  | Quetiapine | Lurasidone |  |
|  | Risperidone/Paliperidone | Opipramol |  |
|  | Trimipramine | Pipamperone |  |
|  | Zuclopenthixol | Promazine |  |
|  |  | Sertindole |  |
|  |  | Sulpiride |  |
|  |  | Tiapride |  |

Psychotropic medications were categorized by their risk of weight according to:

Leucht S, Cipriani A, Spineli L, Mavridis D, Orey D, Richter F, et al. Comparative efficacy and tolerability of 15 antipsychotic drugs in schizophrenia: a multiple-treatments meta-analysis. Lancet. 2013;382:951–62.

Christoph U. Correll, Johan Detraux, Jan De Lepeleire, Marc De Hert. Effects of antipsychotics, antidepressants and mood stabilizers on risk for physical diseases in people with schizophrenia, depression and bipolar disorder. World Psychiatry. 2015

**Supplementary Table 2:** Somatic comorbidities and pharmacological treatments

| **Medical diagnoses** | **ICD-10 codes** | **ATC codes** |
| --- | --- | --- |
| Dysfunction of thyroid‡ | •Hypothyroidism:  E03 Other hypothyroidism  •Hyperthyroidism:  E05 Thyrotoxicosis [Hyperthyroidism] | •Treatments for hypothyroidism:  Levothyroxine sodium (H03AA01)  Associations of levothyroxine and liothyronine (H03AA03)  •Treatments for hyperthyroidism:  Propylthiouracil (H03BA02)  Carbimazole (H03BB01) |
| Chronic obstructive pulmonary disease | J44 Other chronic obstructive pulmonary disease | Indacaterol (R03AC18)  Vilanterol and umeclidinium bromide (R03AL03)  Indacaterol and glycopyrronium bromide (R03AL04)  Tiotropium bromide (R03BB04)  Roflumilast (R03DX07) |
| Cardiovascular diseases | G45 Transient cerebral ischemic attacks and related syndromes  I11 Hypertensive heart disease  I20-I25 Ischemic heart diseases  I60-I69 Cerebrovascular diseases (except I68 Cerebrovascular disorders in diseases classified elsewhere)  I70-I77 Diseases of arteries, arterioles |  |

^‡^ Hypothyroidism was supposed if thyroid-stimulating hormone (TSH) was superior to 10 mUI.L-1 (https://www.revmed.ch/RMS/2010/RMS-273/Les-dysthyroidies-en-medecine-de-premier-recours accession date 24.09.20) whereas hyperthyroidism was attributed if TSH was lower than 0.10 mUI.L-1 (https://www.uptodate.com/ accession date 23.09.20). Other levels of TSH, including a TSH between 5 and 10 mUI.L-1, was considered as if the thyroid function was normal since an increase in TSH level of less than 10 mUI.L-1 was thought to be transient or secondary to other health problems: for example, morbid obesity (https://www.uptodate.com/ accession date 23.09.20). Similarly, TSH between 0.10 and 0.40 mUI.L-1 was considered to be subclinical and treatments are therefore controversial (https://www.uptodate.com/ accession date 23.09.20).

**Supplementary Table 3A:** Definition of metabolic disturbances and metabolic syndrome [International Diabetes Federation (IDF) definition]:

| A. Central obesity | Waist circumference ≥ 94 cm (men) or 80 cm (female) or BMI > 30 kg/m². |
| --- | --- |
| B. Hypertension | Systolic BP ≥ 130 or diastolic BP ≥ 85 mmHg and/or specific treatment for blood pressure abnormality is listed in Supplementary Table 4B. |
| C. Hyperglycemia | Fasting plasma glucose (FPG) ≥ 100 mg/dL (5.6 mmol/L) and/or specific treatment for type 2 diabetes is listed in Supplementary Table 4B. |
| D. Hypertriglyceridemia | Serum triglycerides ≥ 150 mg/dL (1.7 mmol/L) and/or treatment for lipid abnormality is listed in Supplementary Table 4B. |
| E. HDL hypocholesterolemia | HDL cholesterol < 40 mg/dL in men (1.03 mmol/L) or 50 mg/dL in women (1.29 mmol/L) and/or treatment for lipid abnormality is listed in Supplementary Table 4B. |
| Metabolic syndrome | Presence of A plus any two of B, C, D and E. |

BMI=body mass index; cm=centimeters; Kg/m^2^=kilograms per square meter; BP=blood pressure; mmHG=millimeters of mercury; mg/dL=milligrams per deciliter; mmol/L=millimoles per liter; HDL=high-density lipoprotein.

**Supplementary Table 3B:** Treatment used to define hypertension (anti-hypertensive), hyperglycemia (anti-diabetic), and hypertriglyceridemia and HDL hypocholesterolemia (lipid lowering agents)

| **Lipid-lowering agents** | **Anti-diabetic medications** | **Antihypertensives** | | |
| --- | --- | --- | --- | --- |
| Simvastatin (C10AA01)  Pravastatin (C10AA03)  Fluvastatin (C10AA04)  Atorvastatin (C10AA05)  Rosuvastatin (C10AA07)  Pitavastatin (C10AA08)  Bezafibrate (C10AB02)  Gemfibrozil (C10AB04)  Fenofibrate (C10AB05)  Colestyramine (C10AC01)  Ezetimibe (C10AX09)  Simvastatin and ezetimibe (C10BA02) | Human Insulin (A10AB01, A10AC01, A10AD01)  Insulin lispro (A10AB04, A10AD04)  Insulin aspart (A10AB05, A10AD05)  Insulin glulisine (A10AB06)  Insulin degludec and insulin aspart (A10AD06)  Insulin glargine (A10AE04)  Insulin detemir (A10AE05)  Insulin degludec (A10AE06)  Metformin (A10BA02)  Glibenclamide (A10BB01)  Gliclazide (A10BB09)  Glimépiride (A10BB12)  Metformin and  sulfonamides (A10BD02)  rosiglitazone (A10BD03)  sitagliptin (A10BD07)  vildagliptin (A10BD08)  saxagliptin (A10BD10)  linagliptin (A10BD11)  empagliflozin (A10BD20)  Acarbose (A10BF01)  Rosiglitazone (A10BG02)  Pioglitazone (A10BG03)  Sitagliptin (A10BH01)  Vildagliptin (A10BH02)  Saxagliptin (A10BH03)  Linagliptin (A10BH05)  Exenatide (A10BJ01)  Liraglutide (A10BJ02)  Dapagliflozin (A10BK01)  Empagliflozin (A10BK03)  Repaglinide (A10BX02)  Nateglinide (A10BX03)  Desmopressin (H01BA02) | Clonidine (C02AC01)  Moxonidine (C02AC05)  Doxazosin (C02CA04)  Hydrochlorothiazide (C03AA03)  Chlortalidone (C03BA04)  Metolazone (C03BA08)  Indapamide (C03BA11)  Furosemide (C03CA01)  Torasemide (C03CA04)  Spironolactone (C03DA01)  Eplerenone (C03DA04)  Hydrochlorothiazide and potassium-sparing diuretics (C03EA01)  Butizide and potassium-sparing diuretics (C03EA14)  Furosemide and potassium-sparing diuretics (C03EB01)  Oxprenolol (C07AA02)  Propranolol (C07AA05)  Metoprolol (C07AB02)  Atenolol (C07AB03)  Bisoprolol (C07AB07)  Celiprolol (C07AB08)  Nebivolol (C07AB12)  Labetalol (C07AG01)  Carvedilol (C07AG02)  Bisoprolol and thiazides (C07BB07)  Nebivolol and thiazides (C07BB12)  Atenolol and other diuretics (C07CB03)  Metoprolol and other antihypertensives (C07FB02)  Atenolol and other antihypertensives (C07FB03) | Amlodipine (C08CA01)  Felodipine (C08CA02)  Isradipine (C08CA03)  Nifedipine (C08CA05)  Lercanidipine (C08CA13)  Verapamil (C08DA01)  Diltiazem (C08DB01)  Captopril (C09AA01)  Enalapril (C09AA02)  Lisinopril (C09AA03)  Perindopril (C09AA04)  Ramipril (C09AA05)  Quinapril (C09AA06)  Benazepril (C09AA07)  Cilazapril (C09AA08)  Fosinopril (C09AA09)  Captopril and diuretics (C09BA01)  Enalapril and diuretics (C09BA02)  Lisinopril and diuretics (C09BA03)  Perindopril and diuretics (C09BA04)  Ramipril and diuretics (C09BA05)  Quinapril and diuretics (C09BA06)  Cilazapril and diuretics (C09BA08)  Fosinopril and diuretics (C09BA09)  Perindopril and amlodipine (C09BB04)  Ramipril and calcium antagonists (C09BB05)  Trandolapril and calcium antagonists (C09BB10) | Losartan (C09CA01)  Valsartan (C09CA03)  Irbesartan (C09CA04)  Candesartan (C09CA06)  Telmisartan (C09CA07)  Olmesartan medoxomil (C09CA08)  Azilsartan medoxomil (C09CA09)  Losartan and diuretics (C09DA01)  Eprosartan and diuretics (C09DA02)  Valsartan and diuretics (C09DA03)  Irbesartan and diuretics (C09DA04)  Candesartan and diuretics (C09DA06)  Telmisartan and diuretics (C09DA07)  Olmesartan medoxomil with diuretics (C09DA08)  Azilsartan medoxomil and diuretics (C09DA09)  Valsartan and amlodipine (C09DB01)  Olmesartan medoxomil with amlodipine (C09DB02)  Valsartan, amlodipine with hydrochlorothiazide (C09DX01)  Olmesartan medoxomil, amlodipine with hydrochlorothiazide (C09DX03)  Valsartan and sacubitril (C09DX04)  Aliskiren (C09XA02)  Aliskiren and hydrochlorothiazide (C09XA52) |

Defined using: https://icd.who.int/, https://www.whocc.no/atc_ddd_index, and https://compendium.ch/

**Supplementary Table 4.** Independent predictors of psychiatric readmission (univariate analyses)

| *Predictors* | | *Unadjusted Hazard ratio* | *Lower CI 0.95* | *Upper CI 0.95* | *p-value* |
| --- | --- | --- | --- | --- | --- |
| **Sex** (N=16580) | Sex (female) | **0.90** | **0.84** | **0.98** | **0.01** |
|  | sex*time (year) | **1.03** | **1.00** | **1.06** | **0.03** |
| **Age** (N=16580) | Age (≥ 45year) | **0.65** | **0.60** | **0.70** | **<10^-3^** |
|  | Age (≥ 45year)*time (year) | **0.96** | **0.93** | **0.99** | **0.006** |
| **Smoking** (N=10965) | smoking | **1.69** | **1.55** | **1.84** | **<10^-3^** |
|  | smoking*time (year) | 1.00 | 0.97 | 1.04 | 0.85 |
| **Swiss socio-economic position (SSEP)** (N=9937) | SSEP (10units) | **0.96** | **0.93** | **0.99** | **0.02** |
|  | SSEP (10units)*time (year) | 1.00 | 0.99 | 1.02 | 0.87 |
| **HoNOS** (N=5477) | HoNOS at discharge (5units) | **1.18** | **1.14** | **1.22** | **<10^-3^** |
|  | HoNOS at discharge (5units)*time (year) | **0.87** | **0.84** | **0.90** | **<10^-3^** |
| **Suicide attempts** (N=15435) | Suicide attempt | 0.96 | 0.86 | 1.10 | 0.49 |
|  | Suicide attempt*time (year) | 0.97 | 0.88 | 1.04 | 0.56 |
| **Psychiatric diagnosis¹** (N=15435) | Dementia | **0.69** | **0.50** | **0.96** | **0.03** |
|  | Substance abuse | **2.12** | **1.67** | **2.70** | **<10^-3^** |
|  | Psychotic | **2.42** | **1.91** | **3.08** | **<10^-3^** |
|  | Schizoaffective | **2.24** | **1.74** | **2.88** | **<10^-3^** |
|  | Bipolar | **1.88** | **1.46** | **2.41** | **<10^-3^** |
|  | Depression | **1.71** | **1.34** | **2.18** | **<10^-3^** |
| **Psychotropic medication²** (N=16580) | Low risk of weight-gain | 1.10 | 0.98 | 1.24 | 0.11 |
|  | Medium risk of weight-gain | 1.03 | 0.95 | 1.12 | 0.44 |
|  | High risk of weight-gain | 1.08 | 0.92 | 1.21 | 0.13 |
| **BMI** (N=8576) | BMI | **1.01** | **1.00** | **1.02** | **0.01** |
|  | BMI*time (year) | 1.00 | 1.00 | 1.00 | 1 |
| **BMI (WHO)³** (N=8576) | Underweight | 0.90 | 0.76 | 1.06 | 0.20 |
|  | Overweight | 1.08 | 0.99 | 1.18 | 0.10 |
|  | Obese | 1.03 | 0.91 | 1.15 | 0.66 |
| **Central obesity** (N=8666) | Central obesity | 0.96 | 0.88 | 1.05 | 0.36 |
|  | Central obesity*time (year) | 1.02 | 0.97 | 1.08 | 0.38 |
| **Hyperglycemia** (N=11405) | Hyperglycemia | 0.95 | 0.88 | 1.03 | 0.22 |
|  | Hyperglycemia*time (year) | 0.99 | 0.95 | 1.03 | 0.46 |
| **Hypertriglyceridemia** (N=9340) | Hypertriglyceridemia | 0.97 | 0.89 | 1.06 | 0.49 |
|  | Hypertriglyceridemia*time (year) | 1.00 | 0.95 | 1.05 | 0.99 |
| **HDL hypocholesterolemia** (N=10048) | HDL hypocholesterolemia | 0.94 | 0.86 | 1.02 | 0.13 |
|  | HDL hypocholesterolemia*time (year) | 0.99 | 0.95 | 1.04 | 0.75 |
| **Hypertension** (N=5703) | Hypertension | **0.78** | **0.69** | **0.89** | **<10^-3^** |
|  | Hypertension*time (year) | **0.87** | **0.78** | **0.97** | **0.02** |
| **Metabolic syndrome** (N=9362) | Metabolic syndrome | 0.96 | 0.85 | 1.09 | 0.53 |
|  | Metabolic syndrome *time (year) | **1.08** | **1.01** | **1.16** | **0.02** |

Models were conducted using Cox frailty models with time-dependent effects.

¹ Psychiatric diagnoses were based on the ICD-10 classification and were classified as: dementia [F00-F02; G30] | substance use disorders [F10-F19] | psychotic disorders [F20-F24; F28-F29] | schizoaffective disorders [F25] | bipolar disorders [F30-F31] | depression [F32-F33] | others [F03-F09; F34-F99].

² Psychotropic medication was classified according to the risk of weight gain as follows: low risk: amisulpride, aripiprazole, brexpiprazole, chlorprothixene, clotiapine, cariprazine, doxepin, flupentixol, haloperidol, lurasidone, opipramol, pipamperone, promazine, sertindole, sulpiride, tiapride; medium risk: amitriptyline, asenapine, carbamazepine, clomipramine, levomepromazine, lithium, mirtazapine, nortriptyline, pregabalin, paliperidone, quetiapine, risperidone, trimipramine, zuclopenthixol; high risk: clozapine, olanzapine, valproate.

³ Compared to normal weight (Weir CB, Jan A. BMI Classification Percentile And Cut Off Points. [Updated 2022 Jun 27]. In: StatPearls [Internet]. Treasure Island (FL): StatPearls Publishing; 2022 Jan).

Abbreviations: BMI=body mass index; HDL=high-density lipoprotein; HoNOS= Health of the Nation Outcome Scales.

**Supplementary Table 5:** Baseline demographic and clinical characteristics of the readmitted and non-readmitted first-episode psychosis patients

| *Characteristics* | *Total sample*  *285 (100%)* | *Readmission* | | *p- value* |
| --- | --- | --- | --- | --- |
|  |  | *No*  *97 (34%)* | *Yes*  *188 (66%)* |  |
| **Age**, median (IQR), years | 25 (21; 29) | 26 (22; 29) | 25 (21; 29) | 0.28 |
| **Sex**, n (%)  Men  Women | 182 (64)  103 (36) | 60 (62)  37 (38) | 122 (65)  66 (35) | 0.61 |
| **Marital status**, n (%)  Married or registered partnership  Single  Divorced or separated  Widowed | 31 (11)  238 (84)  15 (5)  1 (<1) | 13 (13)  79 (82)  5 (5)  0 (0) | 8 (10)  159 (85)  10 (5)  1 (<1) | 0.69 |
| **Education**, n (%)  Compulsory schooling  No schooling  Apprenticeship  High school  University or college | 10 (32)  2 (7)  9 (29)  4 (13)  6 (19) | 3 (18)  1 (5)  5 (29)  4 (24)  4 (24) | 7 (50)  1 (7)  4 (29)  0 (0)  2 (14) | 0.19 |
| **Employment**, n (%)  Employed  Unemployed  Pensions [disability, retirement]  Other [e.g., student] | 10 (27)  12 (32)  5 (14)  10 (27) | 4 (19)  6 (29)  3 (14)  8 (38) | 6 (38)  6 (38)  2 (12)  2 (12) | 0.31 |
| **Living situation**, n (%)  Home, with others  Home, alone  Homeless  Institutions [(non) medico-social]  Other [prisons, psychiatric clinics, other establishments] | 24 (60)  9 (23)  0 (0)  6 (15)  1 (2) | 17 (77)  3 (13)  0 (0)  1 (5)  1 (5) | 7 (39)  6 (33)  0 (0)  5 (28)  0 (0) | **0.04** |
| **Swiss socio-economic position** median (IQR) | 54 (40; 64) | 53 (39; 63) | 55 (40; 64) | 0.81 |
| **Psychotropic medication by risk of weight gain**, n (%)  No risk  Low risk  Medium risk  High risk | 202 (71)  25 (9)  34 (12)  24 (8) | 68 (70)  8 (8)  14 (15)  7 (7) | 134 (71)  17 (9)  20 (11)  17 (9) | 0.78 |
| **Smokers**, n (%)  No  Yes | 58 (26)  162 (74) | 21 (36)  38 (64) | 37 (23)  124 (77) | 0.06 |
| **Admission status**, n (%)  Voluntary  Compulsory | 16 (39)  25 (61) | 5 (22)  18 (78) | 11 (61)  7 (39) | **0.01** |
| **HoNOS**, median (IQR) | 8 (4; 12) | 8 (4; 12) | 9 (5; 13) | 0.47 |
| **Duration of untreated psychosis**, median (IQR), days | 70 (21; 469) | 57 (19; 439) | 90 (22; 478) | 0.56 |

Pearson’s χ2 test was employed to detect the intergroup difference in terms of categorical variables, while Wilcoxon rank-sum test was used for the continuous variable.

The total n value differs between variable due to missing data.

Number of observations without missing data: age: n=285 (% of missing values: 0%), sex: n=285 (0%), marital status: n=285 (0%); education: n=31 (89%); employment: n=37 (87%); living situation: n=40 (86%); Swiss socio-economic position: n=75 (74%); psychotropic medication: n=285 (0%), smokers: n=220 (33%); admission status: n=41 (86%); HoNOS score: n=210 (26%); Duration of untreated psychosis: n=285 (0%).

Abbreviations: HoNOS= Health of the Nation Outcome Scales; IQR= interquartile range; n= number.

**Supplementary Table 6:** Baseline metabolic characteristics of the readmitted and non-readmitted first-episode psychosis patients at discharge

| *Characteristic* | *Total sample*  *285 (100%)* | *Readmission* | | *p-value* |
| --- | --- | --- | --- | --- |
|  |  | *No*  *97 (34%)* | *Yes*  *188 (66%)* |  |
| **BMI**, median (IQR), kg.m^-2^ | 22.1 (19.6; 24.9) | 21.9 (19.6; 24.8) | 22.2 (19.7; 25.4) | 0.70 |
| A. **BMI categories**, n (%)   - Normal - Underweight - Overweight - Obese | 80 (62)  18 (14)  24 (19)  7 (5) | 27 (73)  3 (8)  6 (16)  1 (3) | 53 (58)  15 (16)  18 (20)  6 (6) | 0.37 |
| B. **Central obesity**, n (%), IDF definition | 10 (8) | 4 (11) | 6 (7) | 0.41 |
| C. **Hypertriglyceridemia**, n (%) | 20 (13) | 10 (16) | 10 (10) | 0.31 |
| D**. HDL hypocholesterolemia**, n (%) | 47 (25) | 17 (24) | 30 (25) | 0.85 |
| E. **Hypertension**, n (%) | 11 (23) | 5 (19) | 6 (27) | 0.51 |
| F. **Hyperglycemia**, n (%) | 23 (10) | 6 (8) | 17 (12) | 0.44 |
| G. **Metabolic syndrome**, n (%), IDF definition | 3 (2) | 2 (3) | 1 (1) | 0.28 |

Pearson’s χ2 test was employed to detect the intergroup difference in terms of categorical variables, while Wilcoxon rank-sum test was used for the continuous variable.

The total n value differs between variables due to missing data.

Number of observations without missing data: BMI: n = 129 (% of missing values: 55%); central obesity: n = 129 (55%); hypertriglyceridemia: n = 159 (44%); HDL hypocholesterolemia: n = 190 (33%); hypertension: n = 48 (83%); hyperglycemia: n = 222 (22%); metabolic syndrome: n= 171 (40%).

A. Defined according to the World Health Organization (WHO) definition: Normal weight (reference): 18.5≤ body mass index (BMI) ≤ 25; Underweight: BMI <18.5 kg.m^-2^; Overweight: 25 ≤ BMI ≤ 30; Obese: BMI>30.

B. Defined using the IDF definition as follows: waist circumference: men ≥ 94 cm; women ≥ 80 cm; or BMI > 30 kg.m^-2^.

C. Defined as follows: systolic BP ≥ 130 or diastolic BP ≥ 85 mm Hg or treatment for hypertension.

D. Defined as follows: fasting plasma glucose ≥ 5.6 mmol/L or treatment for type 2 diabetes.

E. Defined as follows: HDL cholesterol: men < 1.03 mmol/L; women < 1.29 mmol/L or treatment for lipid abnormality.

F. Defined as follows: triglycerides ≥ 1.7 mmol/L or treatment for lipid abnormality.

G. Defined using the International Diabetes Federation (IDF) definition as follows: presence of the B factor plus any two of the following: C / D /E and/or F factors.

Abbreviations: BMI=body mass index; HDL=high-density lipoprotein; IQR= interquartile range; kg=kilograms; m^-2^= square meter; n= number.

**Supplementary Figure 1:** Association between metabolic disturbances and psychiatric readmission considering HoNOS (sensitivity analyses)


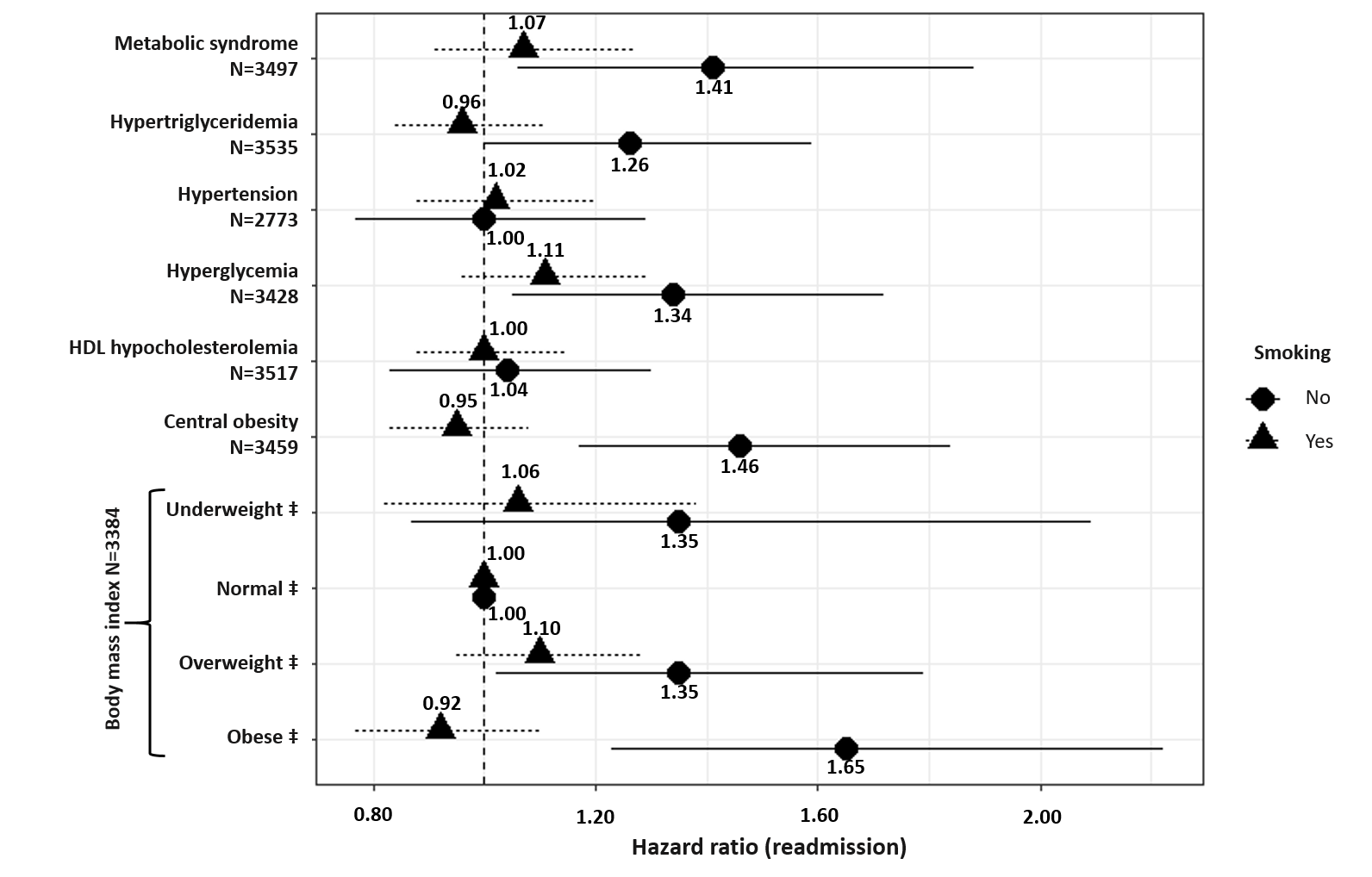


N varies due to missing values.

‡Compared to normal weight and defined according to the World Health Organization (WHO) definition: Normal weight (reference): 18.5 kg.m^-2^≤ body mass index (BMI) ≤ 25 kg.m^-2^; Underweight: BMI <18.5 kg.m^-2^; Overweight: 25 kg.m^-2^≤ BMI ≤ 30 kg.m^-2^; Obese: BMI>30 kg.m^-2^.

Metabolic syndrome was defined using the International Diabetes Federation definition.

Models were adjusted for HoNOS, age, sex, smoking status, psychiatric diagnoses, psychotropic medication, and interaction between the metabolic disturbance analyzed and smoking.

Abbreviations: N: number of observations in the model.

**Supplementary Figure 2:** Association between metabolic disturbances and psychiatric readmission in first-episode psychosis patients (subgroup analyses)


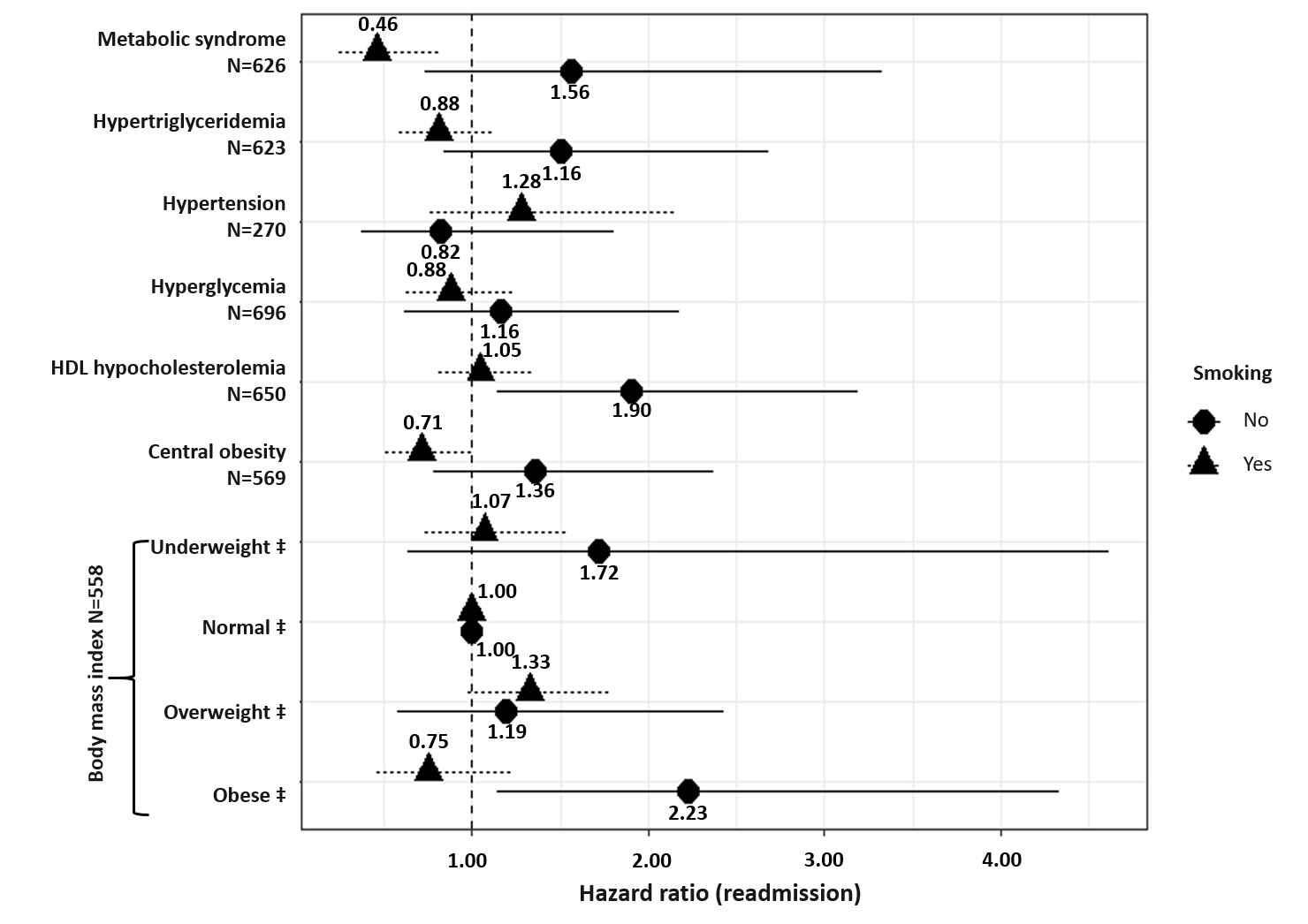


N varies due to missing values.

‡Compared to normal weight and defined according to the World Health Organization (WHO) definition: Normal weight (reference): 18.5 kg.m^-2^≤ body mass index (BMI) ≤ 25 kg.m^-2^; Underweight: BMI <18.5 kg.m^-2^; Overweight: 25 kg.m^-2^≤ BMI ≤ 30 kg.m^-2^; Obese: BMI>30 kg.m^-2^.

Metabolic syndrome was defined using the International Diabetes Federation definition.

Each model was adjusted for age, sex, smoking status, psychotropic medications and duration of untreated psychosis and interaction between smoking and the metabolic disturbances.

Abbreviations: kg=kilograms; m^-2^= square meter; N=number of observations in the model.

**Supplementary Figure 3:** Association between metabolic disturbances and psychiatric readmission in first-episode psychosis patients considering HoNOS (subgroup and sensitivity analyses)


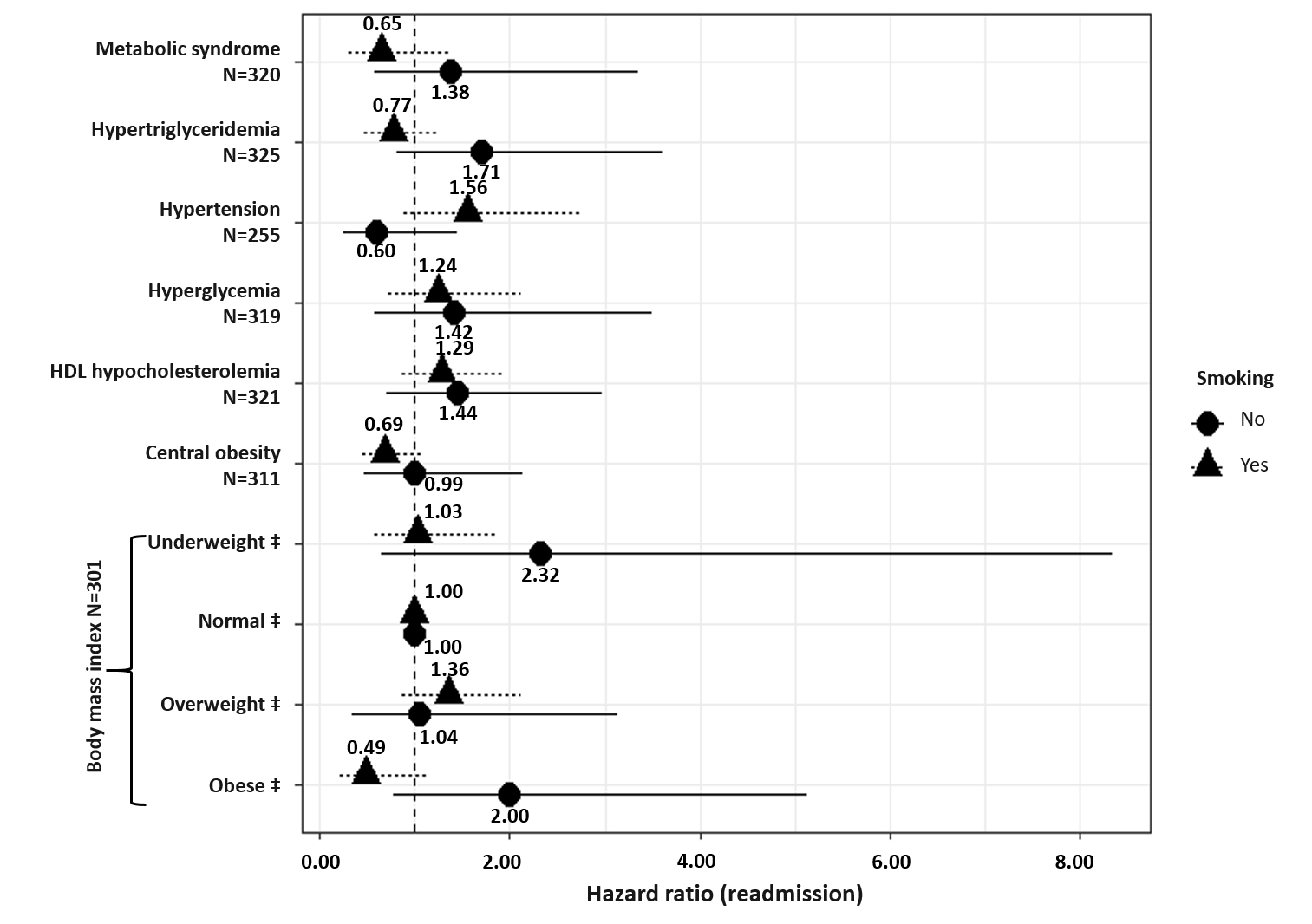


N varies due to missing values.

‡Compared to normal weight and defined according to the World Health Organization (WHO) definition: Normal weight (reference): 18.5 kg.m^-2^≤ body mass index (BMI) ≤ 25 kg.m^-2^; Underweight: BMI <18.5 kg.m^-2^; Overweight: 25 kg.m^-2^ ≤ BMI ≤ 30 kg.m^-2^; Obese: BMI>30 kg.m^-2^.

Metabolic syndrome was defined using the International Diabetes Federation definition.

Each model was adjusted for HoNOS, age, sex, smoking status, psychotropic medications and duration of untreated psychosis and interaction between smoking and metabolic disturbances.

Abbreviations: kg=kilograms; m^-2^= square meter; N=number of observations in the model.
